# A functional proteogenomic analysis of endometrioid and clear cell carcinomas using reverse phase protein array and mutation analysis: protein expression is histotype-specific and loss of *ARID1A*/BAF250a is associated with AKT phosphorylation

**DOI:** 10.1186/1471-2407-14-120

**Published:** 2014-02-22

**Authors:** Kimberly C Wiegand, Bryan T Hennessy, Samuel Leung, Yemin Wang, Zhenlin Ju, Mollianne McGahren, Steve E Kalloger, Sarah Finlayson, Katherine Stemke-Hale, Yiling Lu, Fan Zhang, Michael S Anglesio, Blake Gilks, Gordon B Mills, David G Huntsman, Mark S Carey

**Affiliations:** 1 Biomedical Research Centre, Department of Cellular and Physiological Sciences, University of British Columbia, Vancouver, BC, Canada; 2 Department of Pathology and Laboratory Medicine, University of British Columbia, Vancouver, BC, Canada; 3 Department of Systems Biology, University of Texas, MD Anderson Cancer Center, Houston, TX, USA; 4 Department of Surgical Oncology, British Columbia, Cancer Agency, Vancouver, BC, Canada; 5 Genetic Pathology Evaluation Centre, Vancouver General Hospital, Vancouver, BC, Canada; 6 Department of Bioinformatics and Computational Biology, University of Texas, MD Anderson Cancer Center, Houston, TX, USA; 7 Division of Gynecologic Oncology, University of British Columbia, Vancouver, BC, Canada; 8 Hereditary Cancer Program, British Columbia Cancer Agency, Vancouver, BC, Canada; 9 Department of Medical Oncology, Beaumont Hospital, Dublin, Ireland

**Keywords:** Ovarian cancer, Proteomics, *ARID1A*/BAF250a, *PIK3CA* mutation, AKT, Phosphorylation

## Abstract

**Background:**

Ovarian cancer is now recognized as a number of distinct diseases primarily defined by histological subtype. Both clear cell ovarian carcinomas (CCC) and ovarian endometrioid carcinomas (EC) may arise from endometriosis and frequently harbor mutations in the *ARID1A* tumor suppressor gene. We studied the influence of histological subtype on protein expression with reverse phase protein array (RPPA) and assessed proteomic changes associated with *ARID1A* mutation/BAF250a expression in EC and CCC.

**Methods:**

Immunohistochemistry (IHC) for BAF250a expression was performed on 127 chemotherapy-naive ovarian carcinomas (33 CCC, 29 EC, and 65 high-grade serous ovarian carcinomas (HGSC)). Whole tumor lysates were prepared from frozen banked tumor samples and profiled by RPPA using 116 antibodies. *ARID1A* mutations were identified by exome sequencing, and *PIK3CA* mutations were characterized by MALDI-TOF mass spectrometry. SAM (Significance Analysis of Microarrays) was performed to determine differential protein expression by histological subtype and *ARID1A* mutation status. Multivariate logistic regression was used to assess the impact of *ARID1A* mutation status/BAF250a expression on AKT phosphorylation (pAKT). *PIK3CA* mutation type and PTEN expression were included in the model. BAF250a knockdown was performed in 3 clear cell lines using siRNA to *ARID1A*.

**Results:**

Marked differences in protein expression were observed that are driven by histotype. Compared to HGSC, SAM identified over 50 proteins that are differentially expressed in CCC and EC. These included PI3K/AKT pathway proteins, those regulating the cell cycle, apoptosis, transcription, and other signaling pathways including steroid hormone signaling. Multivariate models showed that tumors with loss of BAF250a expression showed significantly higher levels of AKT-Thr^308^ and AKT-Ser^473^ phosphorylation (p < 0.05). In 31 CCC cases, pAKT was similarly significantly increased in tumors with BAF250a loss on IHC. Knockdown of BAF250a by siRNA in three CCC cell lines wild type for *ARID1A* showed no increase in either pAKT-Thr^308^ or pAKT-S^473^ suggesting that pAKT in tumor tissues is indirectly regulated by BAF250a expression.

**Conclusions:**

Proteomic assessment of CCC and EC demonstrates remarkable differences in protein expression that are dependent on histotype, thereby further characterizing these cancers. AKT phosphorylation is associated with *ARID1A*/BAF250a deficient tumors, however in ovarian cancers the mechanism remains to be elucidated.

## Background

There are important clinical and molecular differences between histological subtypes of ovarian cancer [1,2]. High-grade serous carcinoma (HGSC) is the most common subtype of ovarian cancer representing approximately 70% of cases, whereas clear cell carcinoma (CCC) and endometrioid carcinoma (EC) are less common, occurring at a frequency of approximately 12% and 11% respectively [3]. Whereas HGSC frequently originates from precursor lesions in the fallopian tube [4,5], both CCC and EC may arise from endometriosis and are associated with a younger age of onset [6-10]. Compared to HGSC, both CCC and EC present with earlier-stage disease, and CCC are less sensitive to common chemotherapy regimens used to treat ovarian cancers [11]. Approximately 50% of CCC and 30% of EC harbor mutations in the chromatin-remodeling gene *ARID1A*[12,13]. *ARID1A* functions as a tumor suppressor gene [14], although there are no proven clinical features or differences in outcomes associated with a reduction or loss of expression of its associated protein (BAF250a) by immunohistochemistry (IHC) [12,15].

*PIK3CA* mutations are common in ovarian CCC, endometrial and breast cancers [16-19] and they are frequently co-mutated with *ARID1A* in ovarian CCC [13]. The impact of these co-mutations on phosphorylation of AKT has been studied in endometrial cancers and it was found that tumors with mutated *ARID1A* were associated with increased AKT phosphorylation and pathway activation [20]. In this same study, *PTEN* mutations were also significantly associated with mutations in *ARID1A*. Further research is needed in order to determine whether these observations in endometrial cancer also pertain to ovarian CCC and EC. Therefore, the purpose of this study was to examine protein expression in ovarian EC and CCC using RPPA and to determine whether expression patterns are specific to histotype. We also sought to determine proteomic patterns of expression based on *ARID1A* mutation status/BAF250a protein expression in ovarian EC and CCC, and further study the impact of mutation status (*ARID1A* and *PIK3CA)* and PTEN expression on AKT phosphorylation.

## Methods

### Reverse phase protein array

We performed RPPA on whole tumor lysates as previously described [21,22]. RPPA is suited for functional proteomic assessments of a large number of tumor samples and provides a platform for comparing the relative protein expression between these samples. Using RPPA, 116 antibodies were utilized to assay protein expression of cell surface growth factor receptors, common signaling pathway proteins, steroid hormone receptors, and others proteins involved in proliferation and apoptosis. RPPA provides an estimate of the relative protein expression of each sample in relation to the other samples on the slide. A list of antibodies used for RPPA is included as Additional file 1: Table S1.

### Collection of tumor samples and IHC

Tumor samples (n = 127) were obtained from the gynecologic tumor bank at Vancouver General Hospital and the British Columbia Cancer Agency. The research was conducted with approval from the University of British Columbia institutional review board. Tumor samples were collected at the time of primary surgery and snap frozen within 60 minutes after collection. Patients treated by neoadjuvant chemotherapy were excluded from the study. All patient samples were subjected to pathology review (BG) to confirm the histological subtype and site of origin. Clinical data was accessed through the Cheryl Brown Ovarian Cancer Outcomes Unit and is updated on a regular basis. Tumors were studied by IHC as part of a tumor bank tissue microarray (TMA). Methods for preparation, staining, and scoring of the EOC cases for BAF250a have been previously described [12]; tumors with any degree of nuclear staining for BAF250a protein expression considered a positive score for purposes of this study.

### BAF250a knockdown experiments in clear cell lines

Cell line identities were verified with short tandem repeat (STR) DNA identity testing with the AmpFLSTR® Identifiler® Direct PCR Amplification Kit as per the manufacturer’s protocol (Applied Biosystems®, Life Technologies Corp.) ES2 was obtained from the American Type Culture Collection (ATCC); STR typing to confirm the cell line identity was performed May 12, 2011 and verified against the ATCC STR database. JHOC-5 was obtained from the RIKEN cell bank; STR typing was performed on April 18, 2011 and verified against the RIKEN DSMZ STR Profiler. RMG-1 was obtained from the Health Sciences Research Resources Bank (HSRRB) cell bank; short tandem repeat (STR) DNA identity testing was performed on April 18, 2011 and verified against the HSRRB STR database. Cells were cultured in RPMI with 5-10% FBS, with the exception of the ES-2 cells, which were grown in McCoy’s media. Cells were treated with pooled siRNA (Dharmacon) to *ARID1A* at 20nM using RNAiMax™ as a transfection reagent (Invitrogen) according to the manufacturer’s recommended protocol. Lysates were prepared 60 hours following transfection using Bicine/CHAPS lysis buffer (20 mM Bicine/0.6% CHAPS, pH 7.6). EGF stimulation was performed using 20 ng/ml EGF for 15–20 minutes as a positive control. Protein levels were assessed by Western blotting (SDS-PAGE). Antibodies used were as follows: BAF250a (Sigma- HPA005456), AKTp473 (Cell Signaling 9271, AKTp308 (Cell Signaling 9275), PDK1 (Cell Signaling 3062), p70S6 Kinase (Santa Cruz sc-8418), PTEN (Cell Signaling 9552), and β-actin (Cell Signaling 3700).

### Isoelectric focusing for AKT protein expression

Native capillary isoelectric point focusing was used to assess AKT expression according to recommended protocols using NanoPro™ 2000 (ProteinSimple™, Santa Clara, CA) [23]. G2 premix (pH 5–8) was used for all experiments (ProteinSimple™, # 040–972) Primary antibodies included AKT1 antibody (Santa Cruz, (C-20): sc1618), and ERK 1/2 (ProteinSimple™, Catalog #040-474). Secondary antibodies used were bovine anti-goat (Jackson Labs, Cat# 805-035-180), and goat anti-rabbit (Sigma, #A0545).

### DNA sequencing of tumors and mutational analyses

The *ARID1A* mutations were determined primarily by exome sequencing using next generation technologies and these results were previously described by our group [12]. Common oncogenic mutations were determined by MALDI-TOF mass spectrometry (MassARRAY®, Sequenom Inc.) of primer extension polymerase chain reaction (PCR) products [24]. Mutations of *PIK3CA* were categorized as kinase domain mutation H1047R, and helical domain mutations (E542K, E545K, and also E545D and Q546R). In addition, there were several other less common mutations included (N345K, R88Q, E110K).

### Statistical methods

To assess tumor protein expression unsupervised and supervised cluster analysis was performed using TreeView software (University of Glasgow, Scotland). X-cluster was used to generate heat maps and cluster groups (Stanford). Data was analyzed using SPSS software (Version 20, Chicago, Illinois). Proteins that were differentially expressed in CCC relative to the other subtypes were determined by t-testing and significance analysis of microarray data (SAM) [25]. SAM was used to determine the probability that differences in expression within each group studied are due to false discovery. False discovery rates (FDR) of less 5% were considered significant. Median protein expression levels were used as cut-points for multivariate logistic regression models except for PTEN expression. A cut-point of the lowest 20% of values on RPPA was chosen for PTEN loss based on previous work showing a 28% incidence of PTEN loss in CCC [26], and IHC data from our own center showing a 12% incidence of PTEN loss in 42 cases of CCC/EC.

## Results

### EOC protein expression is histotype specific

**Figure 1:**
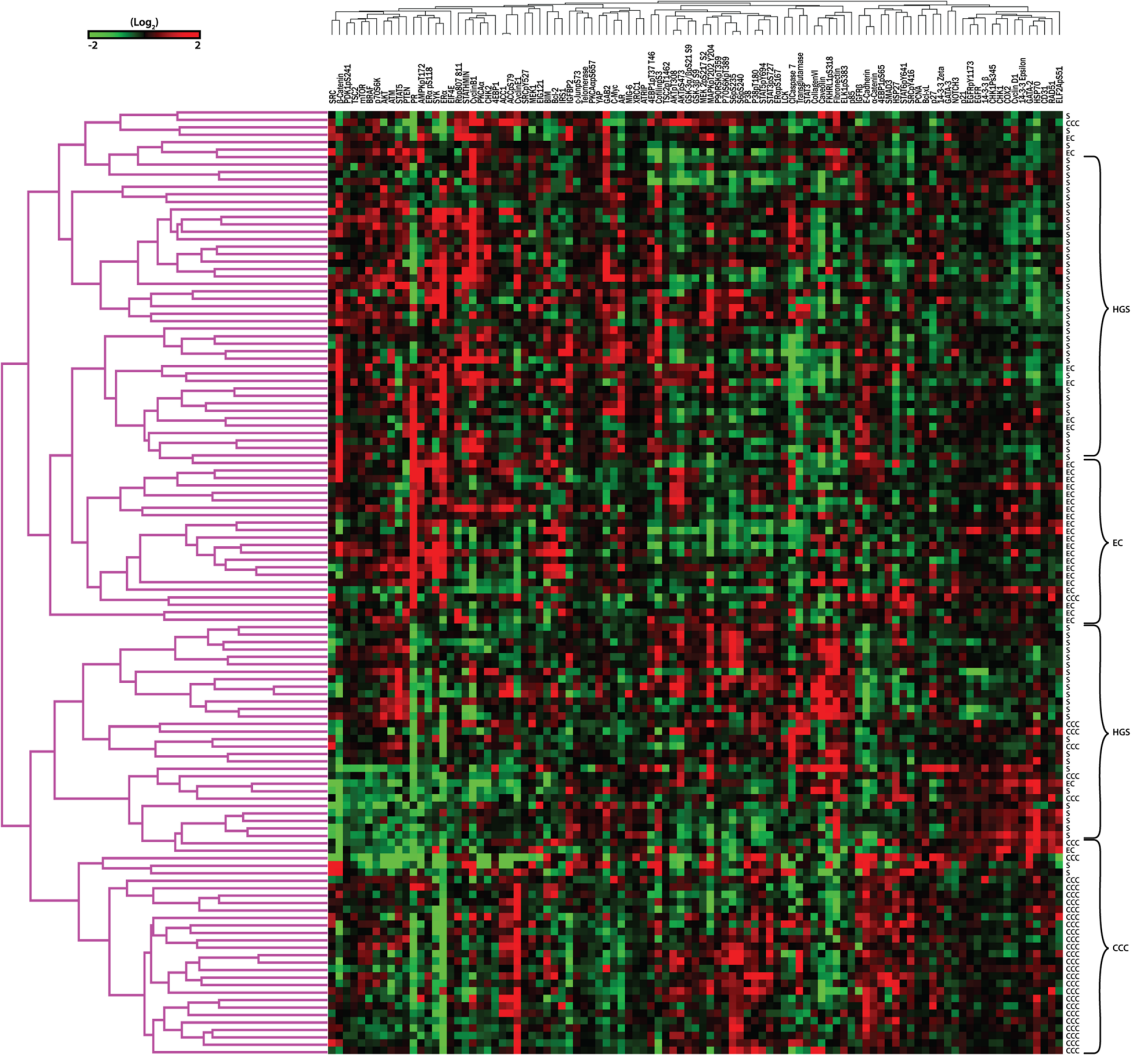
**Hierarchical clustering of samples and proteins analyzed by RPPA.** CCC and ECs form distinct clusters separated by two major high-grade serous carcinoma (HGSC) subgroups. Differential expression of proteins was examined by significance analysis of microarray data (SAM analysis) for both CCC and EC compared to HGSC as the reference group.

**Figure 2:**
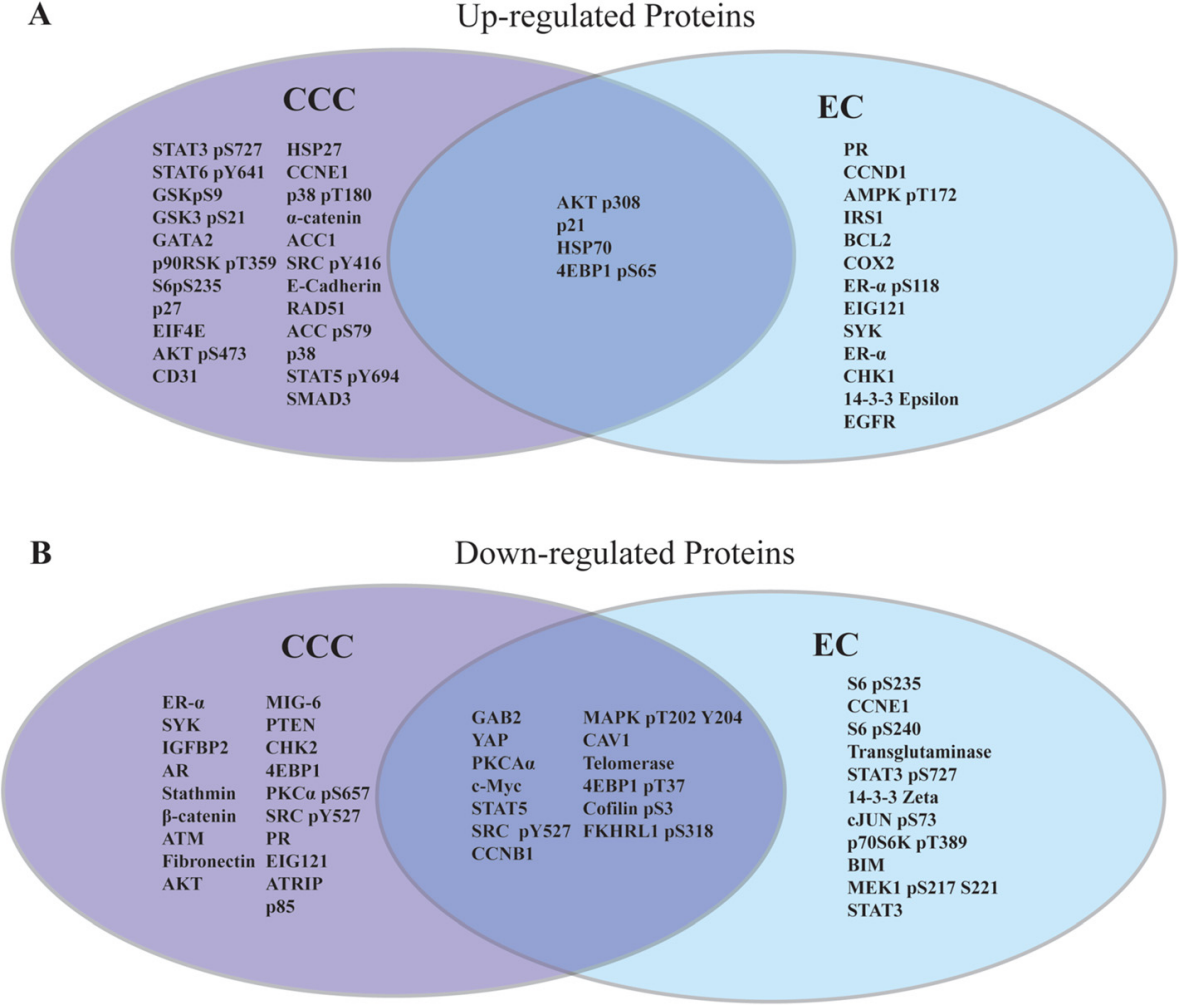
**SAM Analysis of differentially expressed proteins by histotype: A.** Upregulated and **B.** Down-regulated Proteins in Clear Cell and Endometrioid Carcinoma by SAM analysis on RPPA data, with High Grade Serous Carcinoma (HGSC) as the reference group. Proteins with FDR* of <5% are shown. Proteins that are over or under expressed in both CCC and EC are shown in the center between the two subtypes. Note: *FDR (False discovery rate): For each analysis (e.g. CCC vs. EC), the percent probability that the difference in protein expression for each comparison is attributable to chance after correcting for multiple parameter testing.

127 ovarian cancers were submitted for RPPA: 33 CCC, 29 EC, and 65 HGSC. Hierarchical clustering of treatment-naïve samples and proteins analyzed by RPPA is shown in Figure 1. It is evident that clustering is driven by histological subtype; CCC and EC form distinct clusters separated by two major HGSC subgroups. Differential expression of proteins was examined by significance analysis of microarray data (SAM) for both CCC and EC compared to HGSC as the reference group. Figure 2 shows the differentially expressed proteins according to histological subtype. Interestingly a number of proteins are down-regulated in both EC and CCC relative to the HGS group. Notable proteins are GAB2, YAP, Cyclin B1, CofilinpSer^3^, and Caveolin1, 4EBP1pThr^37^, STAT5, and c-Myc. CCC have lower ERα, AR, and PR expression compared to HGSC. The complete list of protein comparisons by SAM and histotype is included as Additional file 2: Table S2. Other proteins with low expression in CCC include β-Catenin, fibronectin, PTEN, 4EBP1, and stathmin. EC are characterized by lower levels of Cyclin E1, phosphorylated S6, 14-3-3 Zeta, BIM, and STAT3.

CCC and EC shared four proteins that were up-regulated compared to HGSC. Notably pAKT-Thr^308^ is higher in both histologies though pAKT-Ser^473^ was of borderline significance. (FDR = 5.2 and 6.9 respectively). Other up-regulated proteins in both CCC and EC include p21, HSP70, and 4EBP1-pSer^65.^ EC as expected have higher expression of ERα, ERαpSer^118^ and PR compared to HGSC, while IRS1, CHK1, EGFR, and Cyclin D1 were also elevated. Some of the up-regulated proteins that characterize CCC include α-Catenin, Cyclin E1, HSP27, E-cadherin, p38, p38pThr^180^Tyr^182^, SMAD3, and GSK proteins.

We then examined differential protein expression associated with BAF250a IHC score by SAM in the 31 CCC with known *ARID1A* mutation status. There were only 2 cases of EC with negative BAF250a IHC so the EC were not included in this analysis. The only differentially expressed protein with a low false discovery rate (FDR) was pAKT-Thr^308^ (FDR < 1%). The top 4 other proteins differentially expressed were as follows: Bcl-2, p27, phosphorylated p38, and pAKT-Ser^473^. FDR rates by SAM for these 5 proteins varied from 16-20%. AKT phosphorylation levels were higher in BAF250a IHC negative tumors while the other 3 proteins had lower levels.

### Investigating the effect of BAF250a/*ARID1A* and *PIK3CA* mutation on protein expression

**Figure 3:**
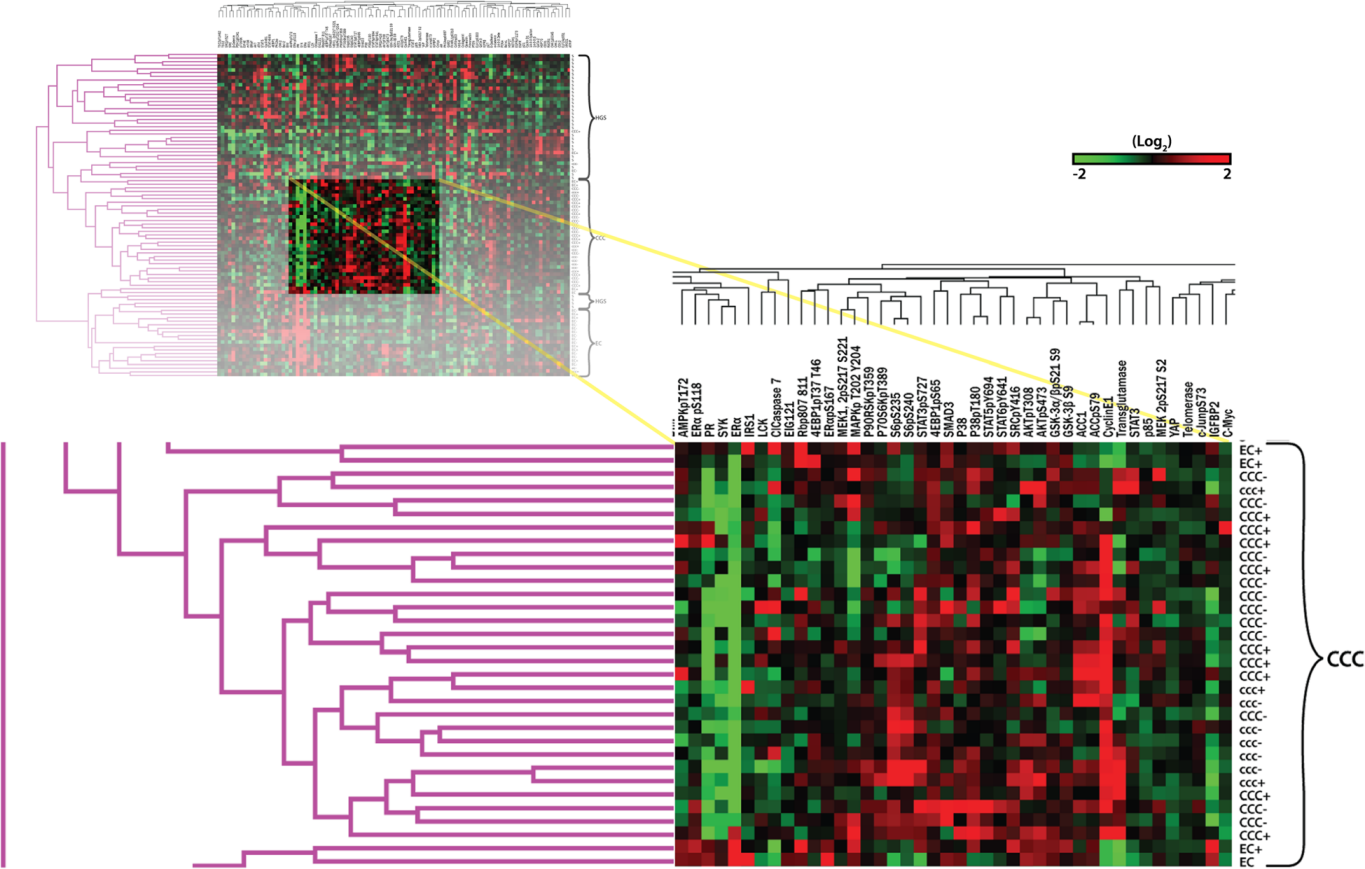
**Heat map with samples categorized according to BAF250a IHC scores and *PIK3CA *mutation status.** No obvious clustering patterns due to either BAF250a by IHC or *PIK3CA* mutation status are present. Note: capital letters for each sample histotype label (eg. CCC) indicates expression of BAF250a on IHC, small letters (e.g. ccc) indicates loss of BAF250a expression. For *PIK3CA* mutations, a + sign after the sample label (e.g. CCC+) indicates the presence of a *PIK3CA* mutation (either helical, kinase, or other) whereas a – sign indicates no mutation.

We examined the effect of *ARID1A* mutation/BAF250a loss on changes in protein expression in the CCC and EC tumors with known *ARID1A* mutation status, BAF250a expression by IHC, and *PIK3CA* mutation status. Table 1 shows the characteristics of this study population. Complete sequencing data for *ARID1A* and *PIK3CA* mutation status, and BAF250a expression by IHC were available on 90 of the 127 original cases used for proteomic analysis. *ARID1A* mutations were present in 17 of 31 CCC in this study (55%), and 5 of 24 (21%) cases of EC. Sequenom MassARRAY® analysis showed *PIK3CA* mutations in 45% of CCC and 46% of EC. Mutations of *PIK3CA* were categorized as kinase domain (H1047R), helical domain (E542K, E545K, E545D and Q546R), or other (less common) including (N345K, R88Q, and E110K). Fifty-seven percent of tumors (13/23) with *ARID1A* mutations had *PIK3CA* mutations versus 38% (12/32) percent of tumors wild type for *ARID1A* (p = 0.3; Chi-square test). Since previous reports have shown that *PIK3CA* mutations have differing effects on *PIK3CA* signaling and AKT phosphorylation, we investigated the role of different *PIK3CA* mutations in relation to *ARID1A* mutation or loss by IHC. While 8/11 (73%) *PIK3CA* helical domain mutants were wild type for *ARID1A;* kinase/other domain mutants were more common in tumors with *ARID1A* mutations (10/14; 71%), (p = 0.05; Fisher’s exact test). Figure 3 shows a heat map with samples categorized according to BAF250a IHC result and *PIK3CA* mutation status. It is evident there are no obvious clustering patterns due either BAF250a or *PIK3CA* mutation status.

**Table 1:** **Patient/sample characteristics with known *ARID1A *** **mutation status according to histotype**

	**Clear cell**	**Endometrioid**	**Serous**
**Characteristic**	**No. (%)**	**No. (%)**	**No. (%)**
**Stage**			
**1/2**	19 (61)	21 (88)	6 (17)
**3/4**	11 (35)	2 (8)	29 (83)
**Missing**	1 (3)	1 (3)	
***ARID1A *** **mutation**			
**No**	14 (45)	19 (79)	35 (100)
**Yes**	17 (55)	5 (21)	0 (0)
***PIK3CA *** **mutation**			
**None**	17 (55)	13 (54)	NT
**Helical**	7 (23)	4 (17)	NT
**Kinase**	4 (13)	4 (17)	NT
**Other**	3 (9)	3 (12)	NT

NT = not tested.

### Loss of BAF250a by IHC, low PTEN expression and activating *PIK3CA* mutations (particularly in tumors without PTEN loss) are associated with elevated pAKT

Table 2 shows the uni- and multivariate analyses of AKT phosphorylation based on *ARID1A* mutation status, BAF250a IHC expression, *PIK3CA* mutation status, and PTEN levels by RPPA. On univariate testing, pAKT-Thr^308^ expression by RPPA was significantly higher in tumors that were BAF250a negative by IHC (p = 0.007), and those with low PTEN (p = 0.025). In contrast, neither *ARID1A* mutation status nor the presence of a *PIK3CA* mutation was associated with a change in pAKT-Thr^308^ levels. Changes in pAKT-Ser^473^ were not associated with any of the above factors on univariate testing.

**Table 2:** **Effect of *PIK3CA *** **mutations, *ARID1A *** **mutations, and BAF250a Expression on Akt phosphorylation**

**Test variable**	**N**	**Univariate testing**		**Multivariate testing**	
		**Mean (SD)**		**Iog**_**e **_**odds ratio/p-value**	
		**pAKT-Thr**^**308**^	**pAKT-Ser**^**473**^	**pAKT-Thr**^**308**^	**pAKT-Ser**^**473**^
***ARID1A *** **mutation**					
**Yes**	22	0.31 (0.64)	0.24 (0.78)	0.41	2.4
**No**	33	0.06 (0.72)	0.0 (0.86)	p = 0.36	p = 0.17
**BAF250a expression**					
**Yes**	43	0.03 (0.67)	0.0 (0.82)	0.03	0.21
**No**	12	0.63 (0.60)*	0.41 (0.83)	p = 0.002*	p = 0.04*
***PIK3CA *** **mutation**					
**Helical**	11	0.11 (0.81)	- 0.25 (0.88)	2.2	1.3
**Kinase**	8	0.50 (0.28)	0.27 (0.39)	p = 0.02*	p = 0.39
**Other**	6	0.39 (0.82)	0.50 (0.98)		
**None**	30	0.05 (0.69)	0.09 (0.86)		
**PTEN level**					
**PTEN low**^**§**^	11	0.78 (0.97)*	0.57 (1.3)	0.1	0.24
**PTEN Normal**	44	0.01 (0.51)	- 0.03 (0.65)	p = 0.01*	p = 0.07

**Note**: univariate mean and standard deviation values represent Iog2 relative protein expression measured by RPPA.

*significant p-value (< 0.05).

**§**lowest 20% of samples by RPPA.

In terms of clarifying the impact of the different *PIK3CA* mutations on pAKT, the distributions (box plots) of pAKT-Thr^308^ and pAKT-Ser^473^ were compared (Additional file 3: Figure S1). By ANOVA, mutation status was not associated with significantly different pAKT levels by RPPA. Helical domain mutants were not associated with an increase in pAKT levels whereas kinase domain and other mutants resulted in slightly higher mean levels of pAKT (both Thr^308^ and Ser^473^) that were not statistically significant. Loss of PTEN is another known mechanism for activation of PI3K pathway signaling. When the eleven low PTEN cases were excluded, kinase domain and other less common mutants had significantly higher pAKT-Thr^308^ (p < 0.05; *t*-test Bonferroni corrected) than wild type, while helical domain mutants did not. For pAKT-Ser^473^, only the uncommon mutants had significantly higher levels by RPPA (p < 0.05; *t*-test Bonferroni corrected) compared to wild type or helical domain mutants.

Multivariate logistic regression showed that pAKT-Thr^308^ levels by RPPA are significantly higher in tumors with loss of BAF250a expression (p = 0.002), those with low PTEN (p = 0.003), and cancers with activating *PIK3CA* mutations (p = 0.02). As with the univariate testing, pAKT-Thr^308^ levels were not associated with *ARID1A* mutation status. On multivariate analysis, increases in pAKT-Ser^473^ were only associated with BAF250a expression levels (p = 0.04). Subgroup analysis of AKT phosphorylation in tumors without *PIK3CA* mutations (18 CCC and 12 EC) showed statistically significant increases in both pAKT-Ser^473^ and pAKT-Thr^308^ for 8 cancers lacking BAF250a expression on IHC (p = 0.05 and 0.008 respectively; *t*-test). Multivariate logistic regression analysis on only the 31 clear cell cancers with known *ARID1A* mutation status showed both pAKT-Thr^308^ and pAKT-Ser^473^ were significantly higher in tumors with BAF250a loss by IHC (p = 0.05 and 0.005 respectively). The EC subgroup was not similarly analyzed, as there were too few *ARID1A* mutant/BAF250a negative cases for comparison.

### siRNA knockdown of BAF250a does not effect AKT phosphorylation in CCC cell lines

**Figure 4:**
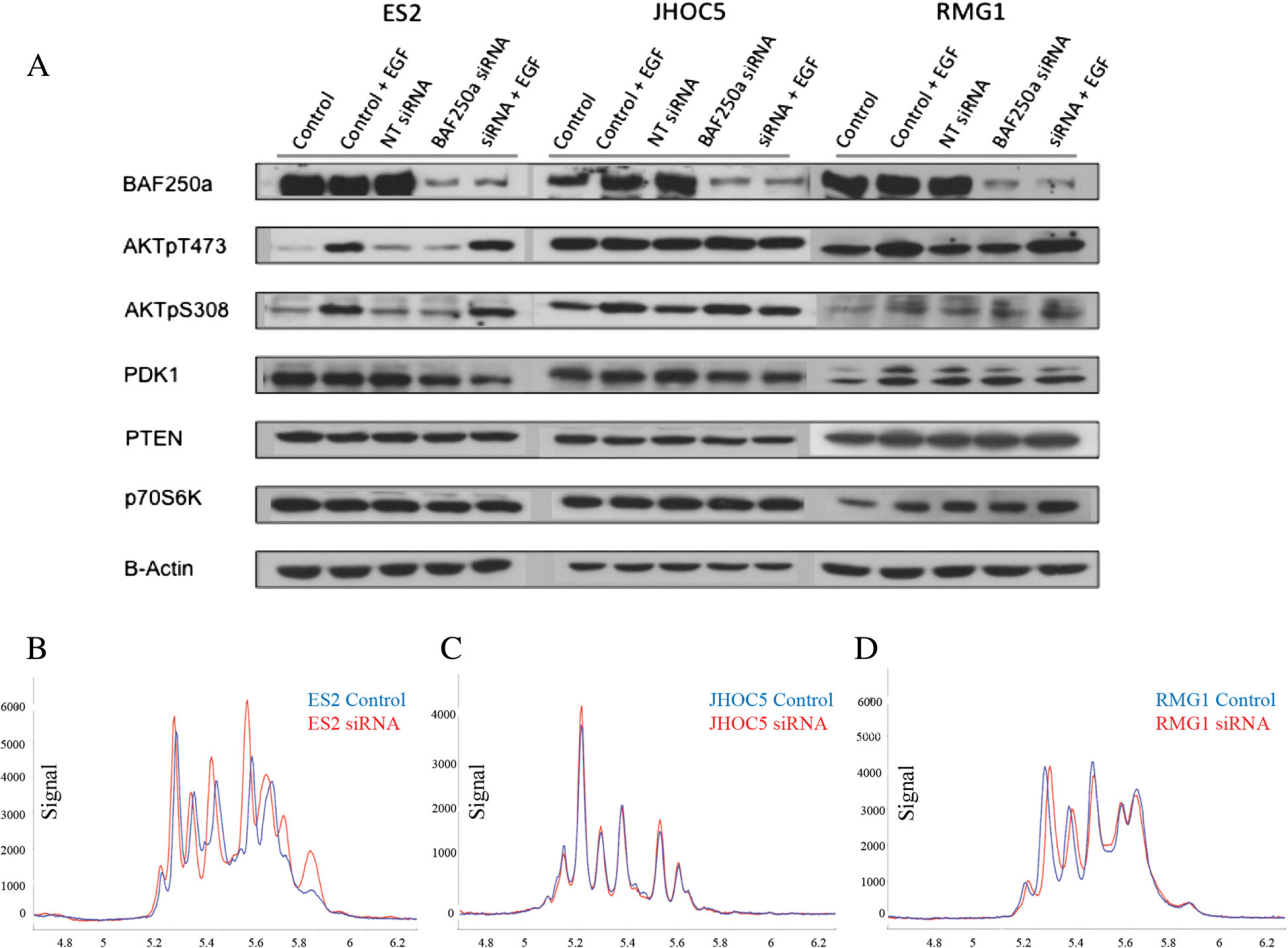
**A) Western blot results from siRNA knockdown of BAF250a on cell lines ES2, JHOC5, and RMG1 to clarify the interaction between BAF250a expression and pAKT.** Despite good knockdown of BAF250a no change in AKT phosphorylation or levels of p70S6K, a downstream signaling protein of pAKT can be seen in the ES2 and RMG1 cell lines. The baseline levels of pAKT are much higher in the JHOC5 cell line, and there is a suggestion of an increase in pAKT-Thr^308^ with BAF250a knockdown without obvious similar changes in pAKT-Ser^473^. PDK1 and PTEN levels did not change with BAF250a knockdown in any of the cell lines. **B-D)** Native protein AKT profiles using capillary tube isoelectric point focusing. Native AKT profiles are consistent with the western blot result in **A**, as little change occurs in AKT/pAKT following siRNA mediated BAF250a knockdown.

In the BAF250a-expressing cell lines RMG1, JHOC5 and ES2, despite good knockdown of BAF250a, changes in AKT phosphorylation (pAKT) or p70S6K, a downstream signaling protein of pAKT were not observed in any of the cell lines tested (Figure 4). Although pAKT-Thr^308^ was difficult to detect in the RMG1 cell lines, with EGF stimulation all cell lines showed an increase in pAKT except for pAKT-Ser^473^ in the JHOC5 cells, however changes in pAKT were not altered by BAF250a knockdown. We also observed that PDK1, PTEN levels did not change with BAF250a knockdown in any of the cell lines. Native protein AKT profiles assessed using capillary tube isoelectric point focusing (Figure 4B-D) are sensitive to phosphorylation events [27]. AKT profiles using this technology confirmed western blot results with no change in AKT profiles in two of the cells lines (RMG1 and JHOC5) and possibly a very slight increase in pAKT (isoforms 1 and 2 for the ES2 cell line). Taken together, these findings indicate that BAF250a knockdown has little to no effect in AKT phosphorylation in the cell lines tested.

### Clinical outcomes

Due to the limited cohort size, survival outcomes were not examined. Instead, FIGO stage distribution at presentation was analyzed to determine if this was influenced by BAF250a loss, *PIK3CA*, or *ARID1A* mutation status. For this comparison, FIGO stage was classified as low-stage (stage 1/2) or advanced-stage (stage 3/4). Those patients with BAF250a loss were more likely to have advanced stage (5/11 = 45% vs. 9/42 = 21%), however this difference was not statistically significant (p = 0.13). Eighty-four percent (21/25) of patients with a *PIK3CA* mutation presented with low-stage disease, compared to 64% (18/28; 2 cases with missing stage) without a mutation. Again, this difference was not statistically significant (p = 0.13). Changes in AKT phosphorylation were also not associated with stage.

## Discussion

We used functional proteomics Reverse Phase Protein Array (RPPA) to assess differential protein expression in CCC and EC compared to HGSC. RPPA has been validated as a means of outcome prediction in breast and ovarian cancers [28,29], and is therefore a useful technology. It is apparent that protein expression is clearly related to histotype, as has been previously shown both by IHC classification and RPPA [1,30]. As expected EC has higher levels of steroid hormone receptor expression (AR, PR) while CCC has lower expression (AR, ER, PR) relative to HGSC. Figure 2 shows that here are more than 50 differentially expressed proteins that characterize EC and CCC from HGSC. It is important to note that our study is descriptive with respect to these protein associations, as they have not been validated by other means.

This study provides evidence that BAF250a expression identifies a subgroup of CCC and EC with higher AKT-Thr^308^ and AKT-Ser^473^ phosphorylation. Based on RPPA expression levels, tumors lacking BAF250a by IHC show higher levels of AKT phosphorylation independent of *PIK3CA* mutation and PTEN loss. Similarly in the cohort of CCC, multivariate analysis showed that increased pAKT (pAKT-Thr^308^ and pAKT-Ser^473^) was associated with BAF250a expression loss. As previously shown in endometrial cancers, increased pAKT was also found in tumors with *ARID1A/*BAF250a loss when both *PIK3CA* mutation or PTEN loss were absent [20].

We previously reported that *ARID1A* mutation status correlates with BAF250a loss by IHC [12]. In this previous report 73% of CCC with an *ARID1A* mutation showed BAF250a loss by IHC, while the correlation was less (50%) in EC. This current study contained a subset of cases from the original report, and found that loss of BAF250a protein by IHC showed a stronger association with pAKT changes than actual *ARID1A* mutation status. Thus, the assessment of BAF250a by IHC may be preferable to sequencing *ARID1A* in tumor samples to identify associations with AKT signaling. Mutations in *ARID1A* are often heterozygous [13] and in such cases it is unknown to what extent BAF250a expression/function is impaired and therefore still detectable by IHC. This could also be true for some somatic mutations. Conversely, IHC can detect epigenetic loss of function events such as methylation that may be as biologically deleterious as homozygous nonsense mutations. These same considerations have previously been described in relation to PTEN assessment. IHC appears to be preferable to sequencing for assessment of PTEN function in endometrial cancers, though a much greater proportion of cases (44%) show PTEN loss by IHC in the presence of a normal PTEN sequence [31]. Further research should be done to confirm the findings of this study and as our sample size is limited due to the fact that CCC and EC are relatively uncommon.

Unlike previously reported observations in endometrial cancers cell lines [20], we have been unable to demonstrate a mechanism by which AKT phosphorylation (pAKT) occurs in tumors using cell line models (RMG1, ES2, and JHOC5). Despite good knockdown of BAF250a in these lines, we could not demonstrate clear increases in pAKT, or downstream signaling. Our findings suggest cell line models may not accurately reflect the signaling changes in tumor samples. Other possible explanations include pAKT modulation by tumor/stromal interactions, cell lineage specificity, or the association of pAKT with other mutations such as *PIK3CA* regulatory domain mutations as they have also been shown to alter pAKT [31].

*PIK3CA* mutations are frequent in EC and CCC, as well as endometrial and breast cancers [16-19]. There is evidence indicating different *PIK3CA* mutations have differing effects on *PIK3CA* signaling and AKT phosphorylation, and therefore may determine prognosis. In breast cancer patients, a recent study suggests patients with *PIK3CA* mutations may actually have a more favorable prognosis [32], and that tumors with *PIK3CA* mutations irrespective of cancer type might have improved response rates to PI3K-directed therapies. In cell lines, helical domain mutants are not consistently associated with increases in pAKT and appear to act through alternate signaling mechanisms potentially involving SGK3 [33]. Similarly, we were not able to identify obvious increases in pAKT signaling in EOC patients with helical domain *PIK3CA* mutants.

This study provides important insights relating to protein expression particularly in ovarian CCC and EC. Because CCC and EC are relatively uncommon compared to high grade serous ovarian cancer (HGSC), it is difficult to gather large numbers of patient samples in order to have the power to detect multiple pathway aberrations. We were unable to show downstream pathway effects resulting from pAKT. Furthermore, selection and validation of antibodies for RPPA may potentially bias results so further study of a larger population of patients is important to confirm and clarify the associations we have found between AKT phosphorylation and BAF250a loss. The identification of downstream signaling aberrations also provides supporting evidence of the biological significance of pAKT. It will be helpful in future studies to also screen for other mutations such as regulatory domain mutations of *PIK3CA* as it may be that *ARID1A* loss is simply associated with other cellular events that increase pAKT rather than being a direct cause.

It is interesting to note that aside from pAKT, proteomic aberrations resulting from *ARID1A*/BAF250a loss in this study are otherwise absent. This finding supports clinical observations to date those patients with *ARID1A* mutant ovarian clear cell endometrial carcinomas do not have a phenotype associated with differing outcomes or treatment responses [12,15,34]. Recently however a report in patients with bladder cancer found that *ARID1A*/BAF250a deficient tumors are associated with a more aggressive phenotype [35]. These findings do not negate the importance of studying signaling pathway aberrations as this information may lead to finding new therapeutic targets. Furthermore, much work is being done to better understand the mechanism of action of novel AKT/PI3K directed therapies [36]. CCC is less sensitive to chemotherapy [37] and the addition of other chemotherapy agents such as irinotecan has been disappointing [38]. While histotype designation is key to classifying ovarian cancers, more research is also needed to find markers that identify patient subgroups who will benefit from novel therapies.

## Conclusions

Using functional proteomics this study identified over 50 proteins that are differentially expressed when CCC and EC are compared to HGSC. Interestingly, functional protein aberrations resulting from the common occurrence of *ARID1A* mutation/BAF250a loss are limited. Only increases in AKT phosphorylation were found to be associated with BAF250a loss though our ability to detect changes in other PI3K pathway proteins may be limited based on sample size. Knockdown experiments in clear cell cancer lines failed to show a direct effect on AKT phosphorylation suggesting an indirect/alternative mechanism for AKT phosphorylation in CCC. Further study is needed to determine the mechanism for the association between BAF250a loss and pAKT in CCC and EC, and explore the diagnostic and therapeutic implications of the described changes in protein expression relating to histotype.

Note: For full protein names see antibody list (Additional file 1: Table S1).

## Abbreviations

ATCC: American type culture collection; CCC: Ovarian clear cell carcinoma; EC: Ovarian endometrioid carcinoma; FDR: False discovery rate; HSRRB: Health sciences research resources bank; HGSC: High-grade serous ovarian cancer; IHC: Immunohistochemistry; MALDI-TOF MS: Matrix-assisted laser desorption/ionization time-of-flight mass spectrometry; pAKT: AKT phosphorylation; PCR: Polymerase chain reaction; RPPA: Reverse phase protein array; SAM: Significance analysis of microarrays; siRNA: Small interfering RNA; STR: Short tandem repeat; TMA: Tissue microarray.

## Competing interests

The authors declare that they have no competing interests.

## Authors’ contributions

Concept and design of study, formulation of research questions: MSC, KCW, BTH, GBM, DGH. Development/application of research methodologies: BG, KS-H, MM, YW, ZJ. YL, BTH, DGH, GBM. Acquisition of data: YL, KS-H, KCW, YW, MSC. Analysis and interpretation of data: FZ, MSC, KCW, SL, SEK, ZJ, BTH. Writing, review and/or revision of the manuscript: MSC, KCW, BG, BTH, GBM, DGH. Study supervision: MSC, DGH, BTH, GBM. All authors approved the final version of the manuscript.

## Authors’ information

KCW was supported by a Dr. Nelly Auersperg Studentship award from the Michael Smith Foundation for Health Research (MSFHR). YW is a recipient of postdoctoral fellowship awards from Canadian Institute of Health Research (CIHR) and MSFHR. GBM and BTH were supported by NCI P50CA083639 and CCSG P30 CA16672. GBM is also supported by the Stand Up to Cancer Dream Team Translational Research Grant, a Program of the Entertainment Industry Foundation (SU2C-AACR-DT0209). BTH is additionally supported by a Career Development Award (CDA) from the Conquer Cancer Foundation of the American Society of Clinical Oncology (ASCO) and a Translational Research Award (TRA) from the Health Research Board (HRB) of Ireland and Science Foundation Ireland (SFI). DGH is supported by the MSFHR and the Dr. Chew Wei Memorial Professorship. MSC was the recipient of a Myer-Levy Fellowship and GBM, BTH, and MSC, were supported by the Kleberg Center for Molecular Markers.

## Pre-publication history

The pre-publication history for this paper can be accessed here:

http://www.biomedcentral.com/1471-2407/14/120/prepub

## Supplementary Material

Additional file 1: Table S1 Antibody list. Click here for file

Additional file 2: Table S2 SAM Data. Click here for file

Additional file 3: Figure S1 Box plots of pAKT levels according to *PIK3CA* Mutation Status. Click here for file
