# Supplementary material for: A functional proteogenomic analysis of endometrioid and clear cell carcinomas using reverse phase protein array and mutation analysis: protein expression is histotype-specific and loss of ARID1A/BAF250a is associated with AKT phosphorylation
Source: BMC Cancer. 2014 Feb 22;14:120. doi: 10.1186/1471-2407-14-120 (PMC3941949; doi:10.1186/1471-2407-14-120)
Supplement: Additional file 1: Table S1 — Antibody list. [file 1471-2407-14-120-S1.pdf]

TABLE S1: ANTIBODY LIST

	Protein	RPPA Abbreviation	Phosphorylation Site	Company	Cat #	RPPA Concentration	Antibody Status**
1	4EBP1	4EBP1		Cell Signaling	9452	1:250	V
2	4EBP1	4EBP1pT37 T46	T37 T46	Cell Signaling	9459	1:500	V
3	4EBP1	4EBP1pS65	S65	Cell Signaling	9456	1:250	V
4	acetyl Co-A carboxylase alpha (1)	ACC1		Epitomics	1768-1	1:300	C
5	acetyl Co-A carboxylase alpha (1)	ACCpS79	S79	Cell Signaling	3661	1:500	V
6	AKT	AKT		Cell Signaling	9272	1:250	V
7	AKT	AKTpS473	S473	Cell Signaling	9271	1:250	V
8	AKT	AKTpT308	T308	Cell Signaling	9275	1:250	V
9	Alpha-catenin*	αCatenin		EMD Millipore	1030	1:3000	V
10	AMPK	AMPK		Cell Signaling	2532	1:250	C
11	AMPK	AMPKpT172	T172	Cell Signaling	2535	1:200	V
12	Androgen Receptor	AR		Epitomics	1852	1:100	V
13	ataxia telangiectasia mutated	ATM		Abcam	32420	1:1000	C
14	ATR interacting protein	ATRIP		Cell Signaling	2737	1:100	C
15	BAD	BADpS112	S112	Cell Signaling	9291	1:200	V
16	Bcl-2	BCL2		Epitomics	1017-1	1:500	V
17	BCL2L1	Bcl-xL		Cell Signaling	2762	1:250	V
18	β-Catenin	B Catenin		Cell Signaling	9562	1:250	V
19	BIM	BIM		Epitomics	1036	1:500	V
20	B-raf	BRAF		Santa Cruz	5284	1:250	C
21	Caspase 7 (cleaved)	ClCasp 7		Cell Signaling	9491	1:250	C
22	Caveolin 1	CAV1		Cell Signaling	3238	1:250	V
23	Collagen VI	Collagen VI		Santa Cruz	20649	1:250	V
24	Cyclin B1	CCNB1		Epitomics	1495	1:500	V
25	Cyclin D1	CCND1		Santa Cruz	718	1:1000	V
26	Cyclin E1	CCNE1		Santa Cruz	247	1:250	V
27	Cyclin E2	CCNE2		Epitomics	1142	1:200	C
28	CD31	CD31		Dako	M0823	1:750	V
29	E-Cadherin	CDH1		Cell Signaling	4065	1:200	V
30	checkpoint kinase 1	CHK1		Cell Signaling	2345	1:100	C
31	checkpoint kinase 2	CHK2		Cell Signaling	3440	1:100	C
32	checkpoint kinase 1	CHK1pS345	S345	Cell Signaling	2348	1:100	C
33	c-Jun	c-Jun		Cell Signaling	9165	1:150	VP

	Protein	RPPA	Phosphorylation	Company	Cat #	RPPA	Antibody
34	c-Jun	c-JunpS73	S73	Cell Signaling	9164	1:100	V
35	C-KIT	c-KIT		Epitomics	1522-1	1:1000	V
36	C-MYC	c-Myc		Cell Signaling	9402	1:100	C
37	Cofilin-1 (non-muscle)	CofilinpS3	S3	Cell Signaling	3313-S	1:500	VP
38	cytochrome c oxidase subunit II	COX2		Epitomics	2969	1:500	C
39	E-Cadherin	Ecadherin		Cell Signaling	3195	1;250	V
40	EGFR	EGFR		Cell Signaling	2232	1:200	V
41	EGFR	EGFRpY1173		Epitomics	1124	1:100	V
42	Eukaryotic translation initiation fact	EIF4E		Cell Signaling	9742	1:200	V
43	Estrogen induced gene	EIG121		Provided by collaborator		1:500	C
44	E74-like factor 2	ELF2ApS51	S51	Cell Signaling	9721S	1:100	VP
45	member of ETS oncogene family	ELK1pS383	S383	Cell Signaling	9181	1:250	C
46	Estrogen Receptor Alpha	ERαp167	S167	Cell Signaling	2514	1:200	VP
47	Estrogen Receptor Alpha	ERαpS118	S118	Epitomics	1091-1	1:300	V
48	Estrogen Receptor Alpha	Erα		Neomarkers	RM-9101-	1:200	V
49	Fibronectin	Fibronectin		Epitomics	1574-1	1:500	V
50	FORTILIN	Fortilin		Provided by R. Chambers		1:5000	C
51	FOXO3A	FKHRL1		Cell Signaling	9467	1:500	VP
52	FOXO3A	FKHRL1pS318 S321	S318 S321	Cell Signaling	2402	1:250	C
53	mTOR	FRAP1		Cell Signaling	2983	1:800	V
54	GRB2-associated binding protein2	GAB2		Cell Signaling	3239	1:500	V
55	GATA-2	GATA-2		Sigma		1:100	VP
56	GATA-3	GATA-3		BD Bioscience	558686	1:100	V
57	GSK-3β	GSK-3β pS9	S9	Cell Signaling	9336	1:500	V
58	GSK-3α/β	GSK-3α/βpS21 S9	S21 S9	Cell Signaling	9331	1:250	V
59	Heat Shock Protein 27	HSP27		Cell Signaling	2402	1:250	C
60	Heat Shock Protein 70	HSP70		Cell Signaling	4872	1:250	C
61	IGFBP2	IGFBP2		Cell Signaling	3922	1:100	V
62	IGFRβ	IGFRβ		Cell Signaling	3027	1:250	C
63	Insuln receptor substrate 1	IRS1		UBI	06-248	1:1000	V
64	lymphocyte-specific protein tyrosin	LCK		Cell Signaling	2752	1:500	C
65	ERK1/ERK2	MAPKpT202 Y204	T202 Y204	Cell Signaling	4377	1:800	V
66	MEK	MEK1		Epitomics	1235-1	1:5000	V
67	MEK	MEK1&2pS217 S221	S217 S221	Cell Signaling	9154	1:1000	V
68	MIG-6 (mitogen-inducible gene	MIG-6		Sigma		1:200	V
69	mTOR	mTOR		Cell Signaling	2983	1:250	V
70	mTOR	mTORpS2448	S2448	Cell Signaling	2971	1:500	C

	Protein	RPPA	Phosphorylation	Company	Cat #	RPPA	Antibody
71	NOTCH3	NOTCH3		Cell Signaling	3268	1:100	C
72	P21	p21		Santa Cruz	397	1:100	V
73	P27	p27		Epitomics	1591	1:200	V
74	P38 MAPK (14)	p38		Cell Signaling	9212	1:100	V
75	p38 MAPK (14)	p38pT180 Y182	T180 Y182	Cell Signaling	9211	1:200	V
76	P53	p53		Cell Signaling	9282	1:3000	C
77	Regulatory subunit PI3-Kinase	p85		UBI	06-195	1:4000	V
78	P70S6K	P70S6K		Epitomics	1494	1:500	V
79	P70S6K	P70S6KpT389	T389	Epitomics	1175-1	1:250	V
80	P90RSK	P90RSKpT359	T359 S363	Cell Signaling	9344	1:500	C
81	PDK1	PDK1		Cell Signaling	3062	1:250	V
82	PDK1	PDK1pS241	S241	Cell Signaling	3061	1;100	V
83	PI3K P110 ALPHA	PI3K P110 ALPHA		Epitomics	1683	1:500	C
84	PROGESTERONE RECEPTOR	PR		Epitomics	1483-1	1:300	V
85	RB	pRbp807_811	S807 S811	Cell Signaling	9308	1:100	V
86	PCNA	PCNA		Abcam	29100	1:1000	C
87	PROGESTERONE RECEPTOR	PR		Epitomics	1483	1:250	V
88	PROTEIN KINASE C ALPHA	PKCAα		Upstate	5-154	1:2000	V
89	PROTEIN KINASE C ALPHA	PKCAαpS657	S657	Upstate(UBI)	06-822	1:2000	C
90	PTEN	PTEN		Cell Signaling	9552	1:300	V
91	RAD51	RAD51		Calbiochem	71	1:250	C
92	S6	S6pS235 S236	S236 S236	Cell Signaling	2211	1:5000	V
93	S6	S6pS240 S244	S240 S244	Cell Signaling	2215	1:2000	V
94	SMAD3	SMAD3		Epitomics	1735-1	1:200	V
95	SRC	SRC		Upstate	05-184	1:200	V
96	SRC	SRCpY416	Y416	Cell Signaling	2101	1:100	C
97	SRC	SRCpY527	Y527	Cell Signaling	2105	1:400	V
98	STAT3	STAT3		Upstate	6-596	1:500	VP
99	STAT3	STAT3pS727	S727	Cell Signaling	9134	1:100	VP
100	STAT3	STAT3pY705	Y705	Cell Signaling	9131	1:500	V
101	STAT5	STAT5		Epitomics	1289	1:250	V
102	STAT5	STAT5pY694	Y694	Epitomics	1208	1:250	VP
103	STAT6	STAT6pY641	Y641	Cell Signaling	9361	1:150	VP
104	STATHMIN	STATHMIN		Epitomics	Jan-72	1:500	V
105	Spleen Tyrosine kinase	SYK		Santa Cruz	1240	1:500	V
106	Telomerase*	Telomerase		SDI	1706	1:250	C
107	TAZ	TAZ		Abcam	3961	1:250	V

	Protein	RPPA	Phosphorylation	Company	Cat #	RPPA	Antibody
108	TRANSGLUTAMINASE	Transglut		Neomarker	MS224	1:750	V
109	TUBERIN	TSC2		Epitomics	1613-1	1:500	V
110	TUBERIN	TSC2pT1462	T1462	Cell Signaling	3617	1:200	V
111	VEGFR2	VEGFR2		Cell Signaling	2479	1:700	V
112	DNA repair protein XRCC1	XRCC1		Cell Signaling	2735	1:100	C
113	YES-ASSOCIATED PROTEIN	YAP		Santa Cruz	15407	1:500	V
114	14-3-3 beta	14-3-3 β		Santa Cruz	628		V
115	14-3-3 epsilon	14-3-3 Epsilon		Santa Cruz	23957		C
116	14-3-3 zeta	14-3-3 Zeta		Santa Cruz	1019		V

* Antibody no longer available

** V=Validated
C=Caution. For further evalutation
VP = Validation pending
