# Supplementary material for: A functional proteogenomic analysis of endometrioid and clear cell carcinomas using reverse phase protein array and mutation analysis: protein expression is histotype-specific and loss of ARID1A/BAF250a is associated with AKT phosphorylation
Source: BMC Cancer. 2014 Feb 22;14:120. doi: 10.1186/1471-2407-14-120 (PMC3941949; doi:10.1186/1471-2407-14-120)
Supplement: Additional file 2: Table S2 — SAM Data. [file 1471-2407-14-120-S2.pdf]

# TABLE S2: SAM analyses

13-02-21

| Histotype | Sample Number |
|---|---|
| HGS | 65 |
| CCC | 33 |
| EC | 29 |

*Analyses Sample Numbers:*

| | n |
|---|---|
| CCC vs. HGS | 98 |
| EC vs. HGS | 94 |

Note: Protein levels are measured relative to HGS protein expression.

**CCC vs HGS** **PROTEINS UP-REGULATED**

| Protein RPPA ID | Score(d) | Numerator(r) | Denominator(s+s0) | Fold Change | FDR(%)* |
|---|---|---|---|---|---|
| HSP27 (c) | 5.413 | 0.866 | 0.16 | 1.822 | 0 |
| 4EBP1pS65 | 5.354 | 0.562 | 0.105 | 1.476 | 0 |
| CCNE1 | 4.671 | 1.039 | 0.222 | 2.055 | 0 |
| p38pT180 | 4.078 | 0.717 | 0.176 | 1.644 | 0 |
| β Catenin | 3.182 | 0.431 | 0.136 | 1.349 | 0 |
| ACC1 (c) | 3.054 | 0.485 | 0.159 | 1.4 | 0 |
| HSP70 (c) | 3.044 | 0.611 | 0.201 | 1.527 | 0 |
| SRCpY416 | 3.019 | 0.494 | 0.164 | 1.409 | 0 |
| Ecadherin | 2.974 | 0.7 | 0.235 | 1.625 | 0 |
| RAD51 (c) | 2.967 | 0.382 | 0.129 | 1.303 | 0 |
| ACCpS79 | 2.612 | 0.443 | 0.17 | 1.359 | 0 |
| p38 | 2.466 | 0.312 | 0.126 | 1.241 | 0 |
| STAT5pY694 (vp) | 2.401 | 0.363 | 0.151 | 1.286 | 0 |
| SMAD3 | 2.394 | 0.354 | 0.148 | 1.278 | 0 |
| p21 | 2.185 | 0.222 | 0.102 | 1.167 | 0 |
| AKTpT308 | 2.168 | 0.364 | 0.168 | 1.287 | 0 |
| STAT3pS727 (vp) | 1.853 | 0.295 | 0.159 | 1.227 | 0 |
| STAT6pY641 (vp) | 1.819 | 0.232 | 0.128 | 1.175 | 0 |
| GSK-3βpS9 | 1.507 | 0.171 | 0.114 | 1.126 | 2.828 |
| GSK3-3α/βpS21 | 1.457 | 0.194 | 0.133 | 1.144 | 2.828 |
| GATA-2 | 1.446 | 0.219 | 0.151 | 1.164 | 2.828 |
| p90RSKpT359 (c) | 1.293 | 0.137 | 0.106 | 1.1 | 5.12 |
| S6pS235 | 1.267 | 0.267 | 0.21 | 1.203 | 5.12 |
| p27 | 1.25 | 0.197 | 0.158 | 1.146 | 5.12 |
| EIF4E | 1.248 | 0.148 | 0.118 | 1.108 | 5.12 |
| AKTpS473 | 1.2 | 0.256 | 0.214 | 1.194 | 5.12 |

| | | | | | |
|---|---|---|---|---|---|
| CD31 | 1.162 | 0.114 | 0.098 | 1.082 | 5.12 |
| S6pS240 | 1.106 | 0.187 | 0.169 | 1.139 | 6.259 |
| EGFRpY1173 | 1.102 | 0.145 | 0.131 | 1.105 | 6.259 |
| Transglut | 1.059 | 0.23 | 0.218 | 1.173 | 6.259 |
| BCL2 | 0.982 | 0.151 | 0.154 | 1.11 | 10.443 |
| EGFR | 0.934 | 0.118 | 0.127 | 1.086 | 10.443 |
| 14-3-3 Epsilon (c) | 0.821 | 0.082 | 0.1 | 1.058 | 15.536 |
| CCND1 | 0.793 | 0.104 | 0.131 | 1.075 | 15.536 |
| 14-3-3 β | 0.679 | 0.08 | 0.117 | 1.057 | 17.435 |
| STAT3 (vp) | 0.622 | 0.092 | 0.148 | 1.066 | 20.929 |

## CCC vs HGS PROTEINS DOWN-REGULATED

| Protein RPPA ID | Score(d) | Numerator(r) | Denominator(s+s0) | Fold Change | FDR(%)* |
|---|---|---|---|---|---|
| AKT | -1.903 | -0.367 | 0.193 | 0.776 | 0 |
| MIG-6 | -1.797 | -0.218 | 0.121 | 0.86 | 0 |
| MAPKpT202 Y204 | -1.656 | -0.39 | 0.235 | 0.763 | 1.116 |
| PTEN | -1.656 | -0.276 | 0.167 | 0.826 | 1.116 |
| CAV1 | -1.629 | -0.538 | 0.33 | 0.689 | 1.116 |
| CHK2 (c) | -1.462 | -0.239 | 0.164 | 0.847 | 2.828 |
| Telomerase (c) | -1.445 | -0.145 | 0.1 | 0.905 | 2.828 |
| 4EBP1 | -1.443 | -0.189 | 0.131 | 0.877 | 2.828 |
| PKCαpS657 (c) | -1.406 | -0.146 | 0.104 | 0.904 | 2.828 |
| SRCpY527 | -1.331 | -0.141 | 0.106 | 0.907 | 2.828 |
| 4EBP1pT37 T46 | -1.295 | -0.213 | 0.164 | 0.863 | 2.828 |
| CCNB1 | -1.266 | -0.396 | 0.313 | 0.76 | 4.079 |
| PR | -1.189 | -0.325 | 0.273 | 0.798 | 4.079 |
| EIG121 (c) | -1.166 | -0.158 | 0.136 | 0.896 | 4.079 |
| ATRIP (c) | -1.162 | -0.14 | 0.12 | 0.908 | 4.079 |
| p85 | -1.161 | -0.141 | 0.122 | 0.907 | 4.079 |
| LCK (c) | -0.972 | -0.146 | 0.15 | 0.904 | 10.443 |
| XRCC1 | -0.93 | -0.095 | 0.102 | 0.936 | 10.443 |
| ClCasp7 (c) | -0.825 | -0.19 | 0.23 | 0.877 | 11.57 |
| Collagen VI | -0.768 | -0.134 | 0.175 | 0.911 | 11.57 |
| p70S6K | -0.73 | -0.116 | 0.158 | 0.923 | 15.536 |
| PCNA (c) | -0.699 | -0.085 | 0.122 | 0.943 | 15.536 |
| PDK1pS241 | -0.676 | -0.077 | 0.114 | 0.948 | 17.435 |
| TSC2 | -0.563 | -0.066 | 0.116 | 0.956 | 20.929 |

## EC vs HGS PROTEINS UP-REGULATED

| Protein RPPA ID | Score(d) | Numerator(r) | Denominator(s+s0) | Fold Change | FDR(%)* |
|---|---|---|---|---|---|
| PR | 7.17 | 2.548 | 0.355 | 5.848 | 0 |
| CCND1 | 3.854 | 0.545 | 0.142 | 1.46 | 0 |
| AMPKpT172 | 3.569 | 0.626 | 0.175 | 1.543 | 0 |
| IRS1 | 3.313 | 0.546 | 0.165 | 1.46 | 0 |
| BCL2 | 2.346 | 0.393 | 0.168 | 1.313 | 0 |

| | | | | | |
|---|---|---|---|---|---|
| COX2 (c) | 2.223 | 0.368 | 0.166 | 1.291 | 0 |
| AKTpT308 | 2.194 | 0.395 | 0.18 | 1.315 | 0 |
| p21 | 1.901 | 0.204 | 0.107 | 1.152 | 1.721 |
| ERαpS118 | 1.807 | 0.302 | 0.167 | 1.233 | 1.721 |
| EIG121 (c) | 1.707 | 0.261 | 0.153 | 1.198 | 2.816 |
| SYK | 1.686 | 0.392 | 0.233 | 1.312 | 2.816 |
| Erα | 1.652 | 0.499 | 0.302 | 1.413 | 2.816 |
| HSP70 (c) | 1.602 | 0.356 | 0.222 | 1.28 | 2.816 |
| CHK1 (c) | 1.507 | 0.182 | 0.12 | 1.134 | 4.891 |
| 4EBP11pS65 | 1.49 | 0.166 | 0.111 | 1.122 | 4.891 |
| 14-3-3 Epsilon(c) | 1.441 | 0.152 | 0.106 | 1.111 | 4.891 |
| EGFR | 1.345 | 0.197 | 0.147 | 1.147 | 4.891 |
| AKTpS473 | 1.287 | 0.296 | 0.23 | 1.228 | 6.864 |
| SRCpY416 (c) | 1.275 | 0.221 | 0.173 | 1.165 | 6.864 |
| α Catenin | 1.23 | 0.166 | 0.135 | 1.122 | 6.864 |
| EGFRpY1173 | 1.15 | 0.162 | 0.141 | 1.119 | 7.228 |
| GSK-3βpS21 | 1.081 | 0.142 | 0.132 | 1.104 | 7.228 |
| ACC1 (c) | 1.064 | 0.164 | 0.154 | 1.12 | 7.228 |
| SMAD3 | 1.004 | 0.138 | 0.138 | 1.1 | 11.616 |
| RAD51 (c) | 0.975 | 0.116 | 0.119 | 1.084 | 11.616 |
| GATA3 | 0.885 | 0.116 | 0.131 | 1.083 | 11.616 |
| GATA2 (vp) | 0.878 | 0.148 | 0.168 | 1.108 | 11.616 |
| IGFRβ (c) | 0.878 | 0.155 | 0.176 | 1.113 | 11.616 |
| EIF4E | 0.851 | 0.108 | 0.127 | 1.078 | 11.616 |
| p38pT180 Y182 | 0.787 | 0.147 | 0.187 | 1.107 | 17.156 |
| ELF2ApS51 (vp) | 0.766 | 0.108 | 0.141 | 1.077 | 17.156 |
| HSP27 (c) | 0.765 | 0.164 | 0.215 | 1.121 | 17.156 |
| ACCpS79 | 0.753 | 0.126 | 0.168 | 1.092 | 17.156 |
| PDK1pS241 | 0.721 | 0.087 | 0.121 | 1.062 | 17.156 |
| STAT6pY641 (vp) | 0.683 | 0.096 | 0.14 | 1.068 | 17.156 |
| CD31 | 0.674 | 0.067 | 0.1 | 1.048 | 17.156 |
| 14-3-3 β | 0.671 | 0.08 | 0.12 | 1.057 | 17.156 |
| ATM (c) | 0.616 | 0.114 | 0.186 | 1.082 | 20.287 |
| SRC | 0.609 | 0.122 | 0.201 | 1.088 | 20.287 |
| BRAF (c) | 0.59 | 0.089 | 0.151 | 1.064 | 20.287 |

## EC vs HGS PROTEINS DOWN-REGULATED

| Protein RPPA ID | Score(d) | Numerator(r) | Denominator(s+s0) | Fold Change | FDR(%)* |
|---|---|---|---|---|---|
| CofilinpS3 (vp) | -3.525 | -0.765 | 0.217 | 0.588 | 0 |
| STAT5 | -3.244 | -0.662 | 0.204 | 0.632 | 0 |
| GAB2 | -3.198 | -0.649 | 0.203 | 0.638 | 0 |
| c-Myc | -2.966 | -0.44 | 0.148 | 0.737 | 0 |
| YAP | -2.78 | -0.308 | 0.111 | 0.808 | 0 |
| S6pS235 | -2.713 | -0.545 | 0.201 | 0.685 | 0 |
| CCNE1 | -2.667 | -0.52 | 0.195 | 0.697 | 0 |
| S6pS240 | -2.644 | -0.408 | 0.154 | 0.754 | 0 |

| | | | | | |
|---|---|---|---|---|---|
| Transglut | -2.635 | -0.54 | 0.205 | 0.688 | 0 |
| 4EBP1pT37 | -2.549 | -0.466 | 0.183 | 0.724 | 0 |
| FKHRL1pS318 (c) | -2.498 | -0.503 | 0.201 | 0.706 | 0 |
| CCNB1 | -2.141 | -0.796 | 0.372 | 0.576 | 0 |
| MAPKpT202.Y204 | -2.113 | -0.539 | 0.255 | 0.688 | 0 |
| STAT3pS727 (vp) | -1.863 | -0.296 | 0.159 | 0.814 | 0 |
| 14-3-3 zeta | -1.797 | -0.245 | 0.136 | 0.844 | 0 |
| cJUNpS73 | -1.765 | -0.212 | 0.12 | 0.864 | 0 |
| p70S6KpT389 | -1.618 | -0.222 | 0.137 | 0.857 | 1.721 |
| BIM | -1.617 | -0.315 | 0.195 | 0.804 | 1.721 |
| MEK1_2pS217.S22 | -1.561 | -0.208 | 0.133 | 0.866 | 1.721 |
| STAT3 (vp) | -1.429 | -0.2 | 0.14 | 0.87 | 2.816 |
| PKCAα | -1.362 | -0.235 | 0.172 | 0.85 | 2.816 |
| SRCpY527 | -1.291 | -0.133 | 0.103 | 0.912 | 4.891 |
| Telomerase (c) | -1.231 | -0.126 | 0.102 | 0.917 | 6.864 |
| p90RSKpT359 (c) | -1.176 | -0.123 | 0.104 | 0.919 | 6.864 |
| MIG-6 | -1.035 | -0.113 | 0.109 | 0.925 | 11.616 |
| CAV1 | -1.001 | -0.362 | 0.362 | 0.778 | 11.616 |
| PCNA (c) | -0.996 | -0.126 | 0.126 | 0.917 | 11.616 |
| MEK1 | -0.86 | -0.137 | 0.16 | 0.909 | 17.156 |
| p85 | -0.808 | -0.098 | 0.121 | 0.934 | 17.156 |
| ATRIP (c) | -0.765 | -0.095 | 0.124 | 0.936 | 20.287 |
| Collagen VI | -0.748 | -0.142 | 0.189 | 0.907 | 20.287 |

Note:
All antibodies validated with the exception
of the following notations:
(c ) = Caution - Further testing pending
(vp) = validation still pending

* FDR = False discovery rate
Within each group, the percent probability that the difference in protein expression for each comparison is attributable to chance after correcting for multiple parameter testing.
