# Supplementary material for: A functional proteogenomic analysis of endometrioid and clear cell carcinomas using reverse phase protein array and mutation analysis: protein expression is histotype-specific and loss of ARID1A/BAF250a is associated with AKT phosphorylation
Source: BMC Cancer. 2014 Feb 22;14:120. doi: 10.1186/1471-2407-14-120 (PMC3941949; doi:10.1186/1471-2407-14-120)
Supplement: Additional file 3: Figure S1 — Box plots of pAKT levels according to PIK3CA Mutation Status. [file 1471-2407-14-120-S3.pdf]

Figure S1: Box plots of pAKT levels according to *PIK3CA* Mutation Status

A: pAKT-Ser473

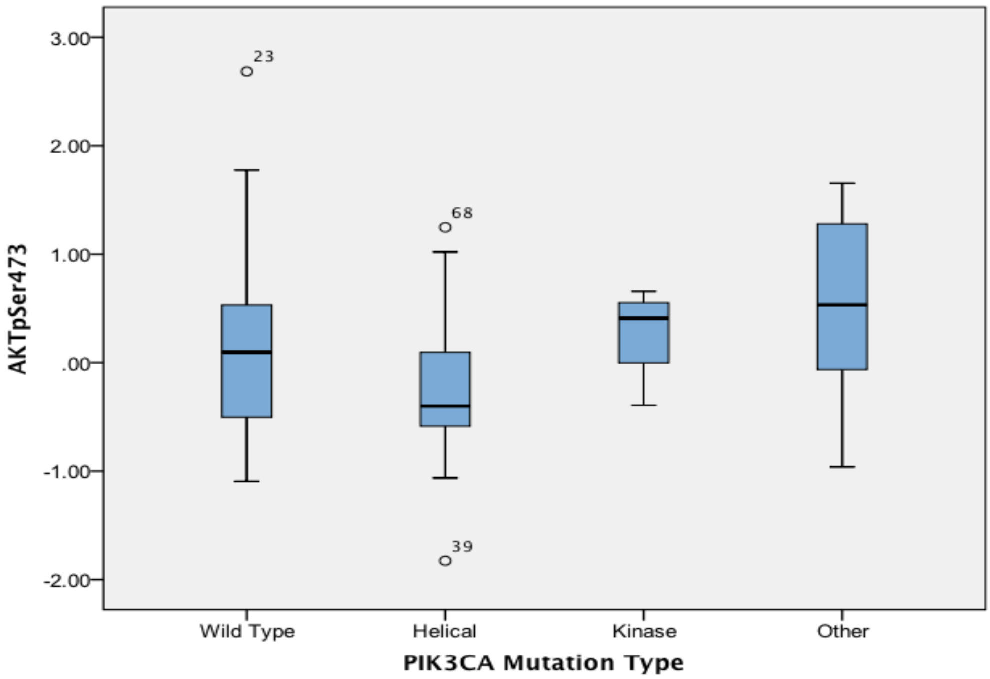

B: pAKT-Thr308

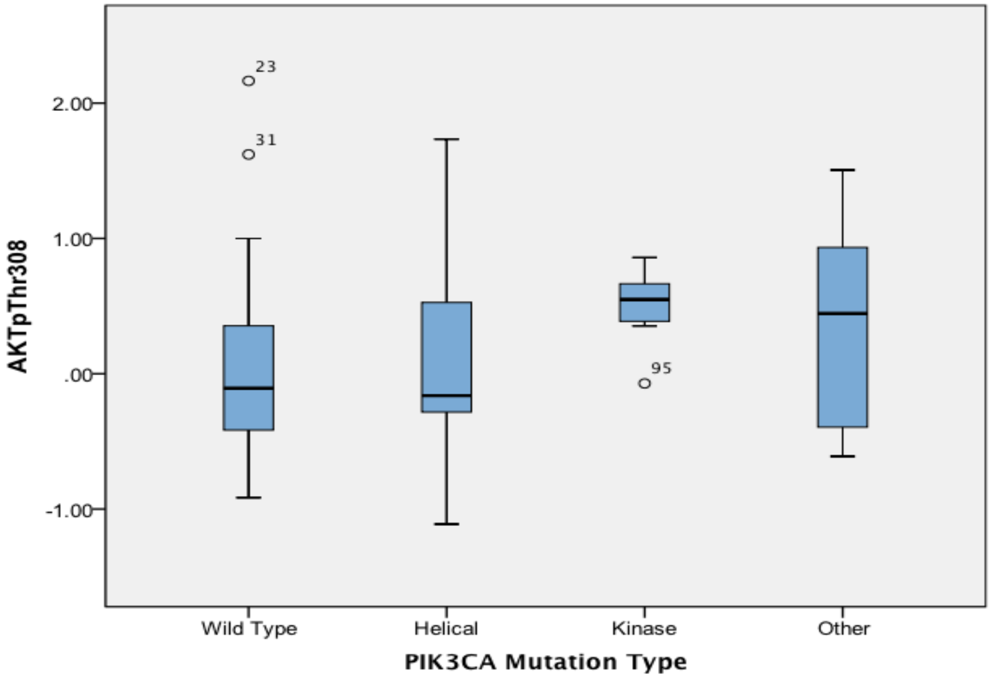

Note: Protein values indicated on Y-axis represent Log(2) relative protein expression as measured by RPPA. Changes in pAKT levels according to *PIK3CA* mutation category were not significantly different (compared to wild type).
